# Sex differentials in the prevalence of behavioral risk factors and non-communicable diseases in adult populations of West Kazakhstan

**DOI:** 10.3389/fpubh.2024.1333887

**Published:** 2024-02-14

**Authors:** Akmaral Baspakova, Anara Zh. Abitova, Nadiar M. Mussin, Asset A. Kaliyev, Gulmira Yerimbetova, Saltanat Zhumagaliyeva, Zhanat Ashimova, Kulyash R. Zhilisbayeva, Aigerim A. Umbetova, Alma U. Nurtazina, Amin Tamadon

**Affiliations:** ^1^Department for Scientific Work, West Kazakhstan Marat Ospanov Medical University, Aktobe, Kazakhstan; ^2^General Surgery, West Kazakhstan Marat Ospanov Medical University, Aktobe, Kazakhstan; ^3^Department of Epidemiology and Biostatistics, Semey Medical University, Semey, Kazakhstan; ^4^Department Natural Sciences, West Kazakhstan Marat Ospanov Medical University, Aktobe, Kazakhstan

**Keywords:** behavioral risk factors, non-communicable diseases, sex differentials, cardiovascular disease, diabetes, hypertension, high cholesterol

## Abstract

**Introduction:**

The prevalence of non-communicable diseases (NCDs) is increasing worldwide. Several modifiable risk factors, such as smoking, alcohol drinking, physical inactivity, and obesity, have been linked to the development of NCDs in both genders. Understanding the prevalence of these risk factors and their associated factors is crucial for effective intervention planning in adult populations. This study aimed to provide an overview of the prevalence and associated factors of these risk behaviors among different genders of adults in West Kazakhstan.

**Methods:**

A cross-sectional study was conducted in four regions of West Kazakhstan. A stratified multistage sampling technique was utilized to obtain a representative sample size of 4,800 participants aged 18 -69 years. Trained researchers administered face-to-face interviews using validated questionnaires to gather information pertaining to sociodemographic characteristics, smoking habits, alcohol drinking, dietary patterns, physical activity levels, body mass index (BMI), and prevalent diseases.

**Results:**

This study, which included 4,800 participants from West Kazakhstan, revealed some striking numerical findings. The overall prevalence rates of behavioral risk factors and metabolic conditions were as follows: smoking was 13.6% (95%CI: 3.2–24.0%), alcohol drinking was 47.0% (27.7–66.3%), current obesity was 22.3% (9.0–35.6%), and physical inactivity was 80.7% (55.4–106.0%). In addition, the overall prevalence rates of metabolic conditions were 25.6% (11.3–39.9%) for hypertension, 7.5% (0.2–15.2%) for diabetes, 11.8% (2.1–21.5%) for high cholesterol, and 13.0% (2.8–23.2%) for cardiovascular diseases. Additionally, a higher prevalence of high cholesterol was observed in men, and a greater prevalence of heart disease was identified in women. Multinomial logistic regression revealed that physical inactivity was associated with hypertension, diabetes, and heart disease, while obesity was linked to hypertension, high cholesterol, and heart disease.

**Discussion:**

This study in West Kazakhstan identified variations in the prevalence of behavioral risk factors and NCDs, highlighting gender, age, and regional disparities. Notably, men showed higher rates of smoking and alcohol drinking, while women exhibited a greater prevalence of physical inactivity and obesity. Gender and regional differences were evident, with the West Kazakhstan region standing out for distinct patterns. Tailored interventions are crucial to address these disparities and enhance public health in the region.

## 1 Introduction

Among middle-aged and older adults, the global concern of lifestyle-related risk factors, including smoking, alcohol drinking, poor nutrition, physical inactivity, and obesity, is paramount (1). Kazakhstan, which is undergoing dynamic societal shifts post-Soviet Union dissolution, experiences challenges linked to urbanization and changing dietary patterns (2). These shifts, notably increased processed food consumption, raise concerns about obesity and non-communicable diseases (NCDs).

Understanding the prevalence of risk factors among middle-aged and older adults in Kazakhstan is imperative due to their contributions to NCDs (3). These factors significantly impact quality of life, functional ability, and independence (4). The country, with its rich cultural heritage and rapid urbanization, faces unique health challenges, including complex dietary practices (2).

Factors such as smoking, excessive alcohol drinking, physical inactivity, and obesity contribute to NCDs in both genders (5). This study aims to systematically investigate these factors among adult populations in West Kazakhstan, focusing on patterns, gender disparities, and regional variations. The objective of this study is to inform targeted public health interventions and improve well-being.

Aligned with the World Health Organization (WHO) recommendations on physical activity and sedentary behavior (WHO guidelines on physical activity and sedentary behavior; WHO: Geneva, Switzerland, 2020), our research explores lifestyle choices, emphasizing sex differentials in middle-aged and older adults in West Kazakhstan.

Kazakhstan’s diverse population and rapid urbanization pose unique health challenges. This study aims to provide a holistic view of health challenges, exploring cultural and socioeconomic determinants influencing behaviors and their impact on the health system.

The study aims to investigate the prevalence and determinants of key behavioral risk factors, such as smoking, alcohol drinking, physical inactivity, and obesity, among adults in West Kazakhstan. By adopting a sex-differential perspective, we aim to uncover patterns and regional variations in these behaviors. This research is significant not only for filling knowledge gaps but also for guiding evidence-based interventions. Our ultimate goal is to promote healthier aging and reduce the burden of NCDs in the region.

## 2 Materials and methods

### 2.1 Ethical issues

The research protocol received ethical approval from the Local Ethics Committee of S.D. Asfendiyarov Kazakh National Medical University, Almaty, Republic of Kazakhstan, under protocol number 12 (118) dated 28 September 2021. Additionally, this study obtained ethical approval from the Central Bioethics Commission of the Ministry of Healthcare of the Republic of Kazakhstan, as indicated by protocol number 14, dated 24 November 2021. The study was duly registered on ClinicalTrials.gov under identifier NCT05122832. All methodologies adhered to relevant ethical and procedural guidelines, and informed consent was appropriately obtained from all study participants or their legal representatives.

### 2.2 Study design and population

This cross-sectional study consisted of a representative sample of people aged 18–69 years in the general population of the population of West Kazakhstan regions for the period October 2021 to May 2022 from four regions.

In this study, a total of 4,800 participants were surveyed, and their characteristics are presented in Tables 1, 2. The participants were categorized based on their regions of residence, and the prevalence rates of various health-related behaviors and conditions were assessed.

**Table 1:** Characteristics of study participants at baseline.

Index	Total	Male	Female
Age groups, *N* (%)			
18–29	674 (14.0)	310 (6.5)	364 (7.6)
30–39	1,021 (21.3)	370 (7.7)	651 (13.6)
40–49	1,094 (22.8)	378 (7.9)	716 (14.9)
50–59	1,205 (25.1)	407 (8.5)	798 (16.6)
60–69	806 (16.8)	258 (5.4)	548 (11.4)
Marital status, *N* (%)			
Single or unmarried	793 (16.5)	308 (6.4)	485 (10.1)
Married	3,419 (71.2)	1,322 (27.5)	2097 (43.7)
Married but living separately	33 (0.7)	10 (0.2)	23 (0.5)
Divorced	293 (6.1)	54 (1.1)	239 (5.0)
Widower/widow	244 (5.1)	22 (0.5)	222 (4.6)
Civil marriage	18 (0.4)	7 (0.1)	11 (0.2)
Ethnic groups, *N* (%)			
Kazakh	3,522 (73.4)	1,291 (26.9)	2,231 (46.5)
Russian	979 (20.4)	328 (6.8)	651 (13.6)
Uzbeks	20 (0.4)	8 (0.2)	12 (0.3)
Ukrainians	50 (1.0)	14 (0.3)	36 (0.8)
Tatars	70 (1.5)	21 (0.4)	49 (1.0)
Other	158 (3.3)	61 (1.3)	97 (2.0)
Education level, *N* (%)			
No schooling	45 (0.9)	30 (0.6)	15 (0.3)
Completed primary school (4 grades)	8 (0.2)	1 (0)	7 (0.1)
Completed secondary school (9 grades)	271 (5.6)	115 (2.4)	156 (3.3)
Completed secondary school (11 grades)	1,121 (5.6)	377 (7.9)	744 (15.5)
Completed high school	2,433 (50.7)	902 (18.8)	1,532 (31.9)
Master’s/Postgraduate/Doctoral studies	922 (19.2)	299 (6.2)	623 (13.0)
Labor force status, *N* (%)			
State employee	648 (13.5)	197 (4.1)	451 (9.4)
Private sector worker	1938 (40.4)	870 (18.1)	1,068 (22.3)
Budget employee	702 (14.6)	188 (3.9)	514 (10.7)
Entrepreneur	296 (6.2)	122 (2.5)	174 (3.6)
Farm worker	14 (0.3)	12 (0.3)	2 (0)
Student	65 (1.4)	38(0.8)	27 (0.6)
Housewife	326 (6.8)	16 (0.3)	310 (6.5)
Pensioner	552 (11.5)	149(3.1)	403 (8.4)
Unemployed (able to work)	220 (4.6)	114 (2.4)	106 (2.2)
Unemployed (unable to work)	39 (0.8)	17 (0.4)	22 (0.5)

**Table 2:** Characteristics of study participants at baseline: behavioral and metabolic risk factors by sex.

Index	Total	Male	Female	*p*-value
Smoking status, *N* (%)				0.001
Yes	655 (13.6)	503 (10.5)	152 (3.2)	
No	4,145 (86.4)	1,220 (25.4)	2,925 (60.9)	
Alcohol drinking, *N* (%)				0.001
Yes	2,254 (47.0)	962 (20.0)	1,292 (26.9)	
No	2,546 (53.0)	761 (15.9)	1785 (37.2)	
Waist circumference, *N* (%)				0.001
< 80 cm	1,649 (34.4)	425 (8.9)	1,224 (25.5)	
80–94 cm	1775 (37.0)	635 (13.2)	1,140 (23.8)	
>94 cm	1,376 (28.7)	663 (13.8)	713 (14.9)	
BMI category, *N* (%)				0.413
Underweight (< 18.5)	95 (2.0)	35 (0.7)	60 (1.3)	
Normal (18.5–24.9)	1806 (37.6)	655 (13.6)	1,151 (24.0)	
Pre-obesity (25.0–29.9)	1831 (38.1)	634 (13.2)	1,197 (24.9)	
Obesity class I (30.0–34.9)	771 (16.1)	277 (5.8)	494 (10.3)	
Obesity class II (35.0–39.9)	227 (4.7)	92 (1.9)	135 (2.8)	
Obesity class III (≥40)	70 (1.5)	30 (0.6)	40 (0.8)	
Physical activity, *N* (%)				0.001
Yes	925 (19.3)	448 (9.3)	477 (9.9)	
No	3,875 (80.7)	1,275 (26.6)	2,600 (54.2)	
Hypertension, *N* (%)				0.001
Yes	1,220 (25.6)	391 (8.1)	837 (17.4)	
No	3,572 (74.4)	1,332 (27.8)	2,240 (46.7)	
High cholesterol, *N* (%)				0.001
Yes	566 (11.8)	157 (3.3)	409 (8.5)	
No	4,234 (88.2)	1,566 (32.6)	2,668 (55.6)	
Diabetes, *N* (%)				0.311
Yes	359 (7.5)	120 (2.5)	239 (5.0)	
No	4,441 (92.5)	1,603 (33.4)	2,838 (59.1)	
Heart disease, *N* (%)				0.389
Yes	623 (13.0)	214 (4.5)	409 (8.5)	
No	4,177 (87.0)	1,509 (31.4)	2,668 (55.6)	

This demographically representative study of the adult populace employed a stratified sampling method, categorized according to Western regions of Kazakhstan, with the primary objective of attaining precise prevalence estimations for health-related indicators across the entirety of West Kazakhstan’s administrative regions. In each regional stratum, a total of 1,200 individuals aged between 18 and 69 years were subjected to examination, spanning four distinct regions: Aktobe region, Atyrau region, Mangistau region, and West Kazakhstan region. The sampling strategy within each stratum (region) adopted a two-tiered cluster sampling framework. In the initial phase, 30 primary sampling areas (PSAs), or clusters, were meticulously chosen. Subsequently, a comprehensive roster of all residents aged 18–69 years, domiciled within the selected clusters, was meticulously compiled. During the second phase, 65 participants were randomly selected from each of these lists, employing a systematic sampling method, while adhering to an anticipated participant outreach rate of 62%. Consequently, this approach resulted in an average of 40 individuals being assessed within each cluster.

### 2.3 Inclusion criteria

This study utilized the WHO STEPS questionnaire to establish specific inclusion criteria, ensuring a comprehensive and representative participant pool. Inclusion criteria encompassed individuals between the ages of 18 and 69 years, spanning both genders. Additionally, participants were required to be residents of the surveyed regions in Kazakhstan, as defined by our research scope (6). No specific inclusion criteria were applied based on gender.

The age range of 18–69 years was chosen to encompass a significant portion of the adult population, allowing for a broad and comprehensive examination of health-related parameters across various age groups within the adult demographic. By including individuals from young adulthood to late middle age, this study could capture a diverse range of health behaviors and conditions.

Individuals within the age range of 18–69 years often experience diverse lifestyle factors. This age range spans crucial life stages where lifestyle choices, such as smoking, alcohol drinking, physical activity levels, and obesity, may significantly impact health outcomes. Exploring these factors in a population with a broad age range provides a more nuanced understanding of how lifestyle choices may vary across different life stages.

The selected age range aligns with the specific focus of the current study, which explores the associations between gender, NCDs, and health-related behaviors. Understanding how these factors interact in adults aged 18–69 years in West Kazakhstan is essential for drawing meaningful conclusions about the prevalence and patterns of smoking, alcohol drinking, physical inactivity, and obesity in this population.

In summary, the rationale behind choosing the age group 18–69 years is rooted in the desire to conduct a thorough and meaningful investigation into the associations between gender, NCDs, and key health-related behaviors in a diverse adult population in West Kazakhstan. This age range allows for a comprehensive exploration of the factors that contribute to health outcomes in the context of the current study.

### 2.4 Exclusion criteria

To maintain the integrity and ethical standards of our study, rigorous exclusion criteria were applied during participant selection.

Individuals lacking the capacity to offer informed consent due to cognitive impairment or any other factor that might undermine their comprehension of the study procedures were excluded.

Non-residents of the surveyed regions were excluded to ensure the geographical relevance of our findings.

Participants who declined to participate in the study were also excluded, as their voluntary participation was fundamental to the research process (6).

### 2.5 Survey

The Russian-translated version of the STEPS questionnaire, which had undergone prior translation (specifically, the WHO STEPS tool, encompassing both basic and advanced modules), was employed for data collection. These WHO STEPS questionnaires were integrated into the HealthTrack mobile application, facilitating their utilization by the certified interviewers who conducted the survey.

### 2.6 Covariates

Blood pressure was measured through three tests, with the caveat that any variation exceeding 10 mm Hg. between readings prompted the utilization of the mean value derived from the two readings closest in proximity. The measurements were taken using an Omron digital automatic blood pressure monitor model HEM-8712, equipped with appropriately sized cuffs, from Omron Health Care Co., Japan28. Elevated blood pressure was defined as systolic blood pressure reaching or exceeding 140 mm Hg. and/or diastolic blood pressure equal to or exceeding 90 mm Hg. during the study or as per previously established diagnoses of arterial hypertension.

Diabetes was defined by fasting plasma glucose levels of ≥7.0 mmol/L (126 mg/dL), 2 h plasma glucose levels of ≥11.1 mmol/L (200 mg/dL), HbA1c concentrations of ≥6.5%, a self-reported history of diagnosed diabetes, or the use of anti-diabetic medications. High cholesterol was determined by fasting serum total cholesterol levels of ≥6.22 mmol/L or the utilization of cholesterol-lowering medications.

Body mass index (BMI) indicators were categorized into five distinct groups: BMI < 18.4—indicative of underweight; BMI ≥ 18.5 and < 24.9—reflecting normal weight; BMI ≥ 25 and < 29.9—indicative of overweight; BMI ≥ 30 and < 34.9—representing obesity of the first degree; and BMI > 35—characterizing obesity of the second degree (7). The BMI was calculated using a standardized protocol based on the WHO guidelines. Weight and height measurements were obtained using calibrated instruments, ensuring accuracy and reliability in the data collection process. Trained personnel conducted these measurements following established procedures to minimize potential sources of error. The BMI of the study participants was calculated using precise anthropometric measurements. Trained personnel conducted height and weight measurements during face-to-face interviews. Height was measured to the nearest 0.1 cm using a standard height meter, and weight was measured to the nearest 0.1 kg using calibrated electronic scales. The measurements followed standardized procedures, and participants were asked to remove heavy outer clothing and shoes before measurements. Consistent measurement instruments were utilized across all study locations. The same model of calibrated electronic scales and height meters was employed to maintain measurement uniformity and accuracy. The instruments were regularly calibrated to ensure the reliability of the measurements. No declared values were used for the calculation of BMI. All measurements were taken directly from the study participants during the data collection process. To ensure the accuracy and reliability of the collected data, quality control measures were implemented, including training sessions for data collectors, regular calibration of instruments, and periodic checks on measurement procedures.

In this study, cardiovascular disease is operationally defined to encompass myocardial infarction, stroke, and coronary artery disease. The term cardiovascular disease refers to a class of diseases that involve the heart or blood vessels. In the context of the study, the term “cardiovascular disease” is a broad category that includes various conditions affecting the heart and blood vessels. Myocardial infarction is commonly known as a heart attack, which occurs when the blood supply to a part of the heart muscle is blocked, leading to damage or death of the heart tissue (8). Stroke refers to the condition where there is a sudden interruption in the blood supply to the brain, leading to damage or death of brain cells. Strokes can be caused by a blockage or bleeding in the brain (9). Coronary artery disease is a condition where the blood vessels supplying the heart muscle (coronary arteries) become narrow or blocked, affecting blood flow to the heart (10).

With regard to educational attainment, survey respondents were categorized into the following groups: those with no formal schooling; individuals who had completed primary education (up to grade 4); those who had completed secondary education (up to grade 9); those with a secondary education diploma (up to grade 11); individuals with higher education qualifications; respondents engaged in master’s, postgraduate, or doctoral studies; and those who declined to provide an answer.

Based on nationality, respondents were segmented into distinct groups encompassing Kazakhs, Russians, Uzbeks, Ukrainians, Uighurs, and Tatars, individuals belonging to other ethnic backgrounds, and those who chose not to disclose their ethnicity.

Marital status was classified into the following categories: single or unmarried, married, individuals in a married or married but living separately status, divorced, widowed, individuals in a civil partnership, and individuals who declined to respond.

With respect to smoking habits, respondents were categorized into two groups, distinguishing between smokers and non-smokers. Smoking involves inhaling tobacco smoke from cigarettes, cigars, or pipes.

In terms of alcohol drinking, the questionnaire encompassed a series of inquiries designed to ascertain the respondent’s alcohol drinking habits, specifically determining whether they engage in alcohol drinking. Alcohol drinking is defined as the consumption of beverages containing ethanol, such as beer, wine, and spirits.

According to the WHO guidelines on physical activity and sedentary behavior, adults should engage in at least 150–300 min of moderate-intensity aerobic physical activity or at least 75–150 min of vigorous-intensity aerobic physical activity, or an equivalent combination of moderate- and vigorous-intensity activity throughout the week, to achieve substantial health benefits (11).

Dietary habits were evaluated through a series of inquiries, including questions such as “On an average day, how many servings of fruit do you typically consume?” and “On an average day, how many servings of vegetables do you typically consume?” The participants’ responses were categorized into quartiles. Respondents indicated their customary consumption of standard-sized food portions, with a maximum frequency of up to six times per day. The frequency and quantity of each specific food item, encompassing both fruits and vegetables, were then converted into an average daily intake for each respective category (12).

### 2.7 Statistical analysis

All statistical analyses were weighted to ensure the representation of the entire adult population in West Kazakhstan, aged 18–69 years, by employing individual sampling weights to account for non-response. Prior to proceeding with further analyses, an assessment of the normality of the variables was conducted. Comparative analysis of participant characteristics across various residential settings was executed through chi-square tests applied to categorical variables. The study calculated the overall prevalence of smoking, alcohol drinking, physical inactivity, and obesity. Additionally, the prevalence of individual behavioral risk factors was delineated by gender, age, and the regions of West Kazakhstan, namely, Aktobe region, Atyrau region, Mangystau region, and West Kazakhstan region. To visually depict the geographical distribution of these behavioral risk factors, the province-specific prevalence was graphically represented using GraphPad Prism 7.0.

To investigate the connection between behavioral risk factors and metabolic conditions, binary logistic regressions and multinomial logistic regressions were employed to derive odds ratios (ORs) and corresponding 95% confidence intervals (CIs). In the binary logistic regression models, metabolic conditions, categorized as either present or absent, were designated as dependent variables, while behavioral risk factors served as the independent variables. Statistical significance was established at a threshold of *p*-values below 0.05.

It is pertinent to clarify that gender-specific analyses were undertaken, encompassing separate analyses for men and women. This approach was adopted to discern potential sex-specific patterns and variations in the relationship between behavioral risk factors and metabolic conditions. The distinction between gender-specific analyses ensures a nuanced understanding of these associations, acknowledging potential differences in health behaviors and outcomes between men and women.

All statistical analyses were conducted using SPSS, specifically version 25.0, developed by IBM SPSS, Inc. and headquartered in Chicago, Illinois, United States.

## 3 Results

### 3.1 Characteristics of study participants

Tables 1, 2 display the characteristics of the study participants (*N* = 4,800), categorized by their respective regions of residence. The overall prevalence rates of smoking, alcohol drinking, current obesity, and physical inactivity were 13.6% (95%CI: 3.2–24.0%), 47.0% (27.7–66.3%), 22.3% (9.0–35.6%), and 80.7% (55.4–106.0%), respectively. Additionally, the overall prevalence rates of hypertension, diabetes, high cholesterol, and cardiovascular diseases were 25.6% (11.3–39.9%), 7.5% (−0.2 to 15.2%), 11.8% (2.1–21.5%), and 13.0% (2.8–23.2%), respectively.

Based on the data presented in Table 3, it is evident that a substantial majority of respondents, constituting 80.4% (95%CI: 55.1–105.7%), reported the consumption of one daily serving of fruit. However, when categorized by gender, only 29.7% (95%CI: 14.3–45.1%) of men and 50.7% (95%CI: 30.6–70.8%) of women reported the same dietary habit. Similarly, responses regarding the consumption of one daily serving of vegetables were prevalent, with 73.0% (95%CI: 48.9–97.1%) of respondents indicating this practice. When stratified by gender, 26.8% (95%CI: 12.2–41.4%) of men and 46.2% (95%CI: 27.0–65.4%) of women reported consuming one daily serving of vegetables.

**Table 3:** Characteristics of nutrition of study participants at baseline.

Index	Total	Male	Female	*p*-value
How many servings of fruit do you consume on one of these days?				0.015
Q1^a^	3,858 (80.4)	1,424 (29.7)	2,434 (50.7)	
Q2	440 (9.2)	139 (2.9)	301 (6.3)	
Q3	334 (7.0)	113 (2.4)	221 (4.6)	
Q4	168 (3.5)	47 (1.0)	121 (2.5)	
How many servings of vegetables do you consume on one of these days?				0.211
Q1	3,505 (73.0)	1,288 (26.8)	2,217 (46.2)	
Q2	726 (15.1)	250 (5.2)	476 (9.9)	
Q3	243 (5.1)	84 (1.8)	159 (3.3)	
Q4	287 (6.0)	87 (1.8)	200 (4.2)	
Q5	39 (0.8)	14 (0.3)	25 (0.8)	
How often do you add salt or savory sauces to food before eating it or directly during meals?				0.001
Always	879 (18.3)	290 (6.0)	589 (12.3)	
Often	678 (14.1)	281 (5.9)	397 (8.3)	
Sometimes	1,258 (26.2)	510 (10.6)	748 (15.6)	
Rarely	1,107 (23.1)	410 (8.5)	697 (14.5)	
Never	878 (18.3)	232 (4.8)	646(13.5)	
How often are salt, salty spices, or salty sauces added during cooking in your household?				0.001
Always	1979 (41.2)	633 (13.2)	1,346 (28.0)	
Often	690 (14.4)	272 (5.7)	418 (8.7)	
Sometimes	1,142 (23.8)	444 (9.3)	698 (14.5)	
Rarely	824 (17.2)	318 (6.6)	506 (10.5)	
Never	165 (3.4)	56 (1.2)	109 (2.3)	
How often do you eat processed foods high in salt?				0.001
Always	387 (8.1)	136 (2.8)	251 (5.2)	
Often	895 (18.6)	373 (7.8)	522 (10.9)	
Sometimes	1773 (36.9)	656 (13.7)	1,117 (23.3)	
Rarely	1,493 (31.1)	495 (10.3)	998 (20.8)	
Never	252 (5.3)	63 (1.3)	189 (3.9)	
How much salt or salty sauces do you think you consume?				0.001
Always	81 (1.7)	28 (0.6)	53 (1.1)	
Often	475 (9.9)	200 (4.2)	275 (5.7)	
Sometimes	3,116 (64.9)	1,156 (24.1)	1960 (40.8)	
Rarely	888 (18.5)	267 (5.6)	621 (12.9)	
Never	240 (5.0)	72 (1.5)	168 (3.5)	
How important is it for you to reduce salt in your diet?				0.001
Always	2,138 (44.5)	702 (14.6)	1,436 (29.9)	
Often	2,262 (47.1)	861 (17.9)	1,401 (29.2)	
Sometimes	400 (8.3)	160 (3.3)	240 (5.0)	
Do you think that eating too much salt or savory sauces can cause you serious health problems?				0.001
Always	3,285 (68.4)	1,087 (22.6)	2,198 (45.8)	
Often	1,109 (23.1)	465 (9.7)	644 (13.4)	
Sometimes	406 (8.5)	171 (3.6)	235 (4.9)	

a “Q” represents the quartiles (Q1–Q4) of reported servings of fruit consumed on one of these days. For each quartile, the table provides the total number and percentage of participants, as well as the gender-specific distribution. Quartiles are a statistical method of dividing a data set into four equal parts, offering insights into the distribution of fruit consumption within the study population.

Regarding the consumption of processed foods characterized by elevated salt content, it was observed that 26.7% (95%CI: 12.1–41.3%) of respondents reported frequent consumption. However, when gender-specific differences were considered, 10.9% (95%CI: 1.6–20.2%) of men and 16.1% (95%CI: 4.8–27.4%) of women reported a similar dietary pattern.

### 3.2 Gender-related prevalence of smoking, alcohol drinking, physical inactivity, and obesity in West Kazakhstan

**Figure 1:**
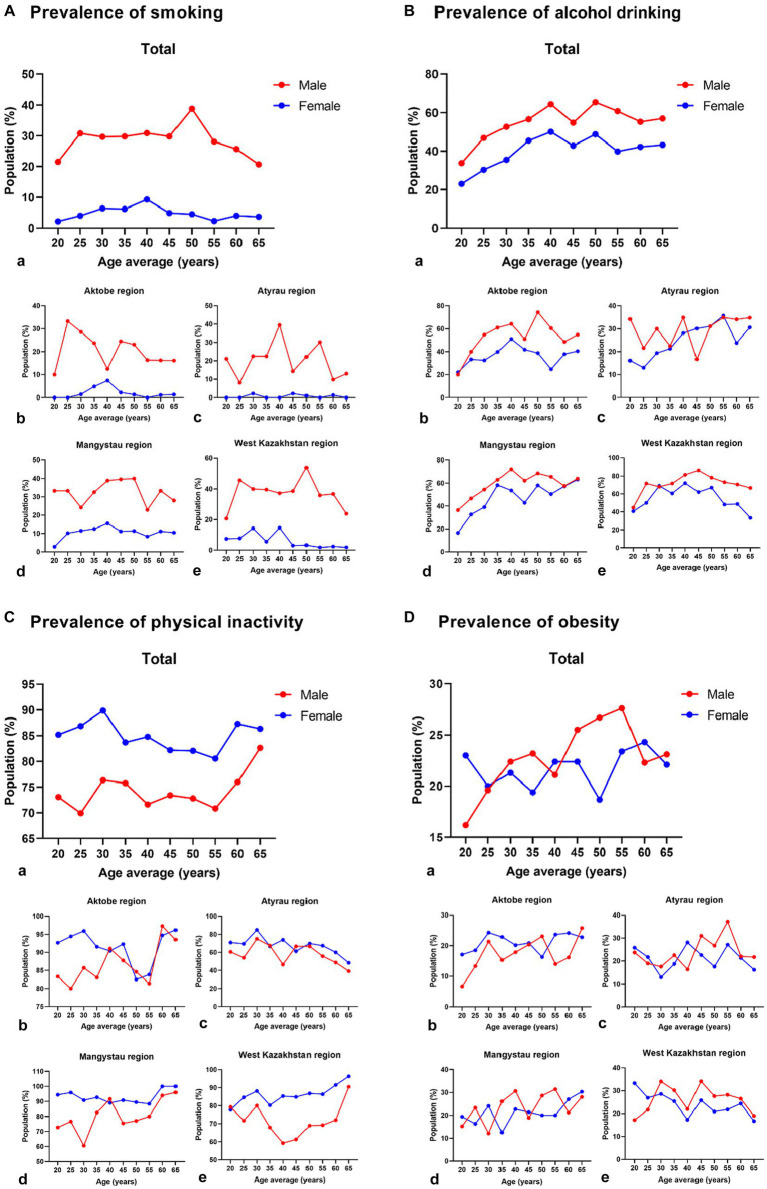
Prevalence of current smoking, alcohol drinking, physical inactivity, and obesity among men and women in the regions of West Kazakhstan. a–e Graphs with small letters labelling show total and four regions of west Kazakhstan.

Figure 1 illustrates the age-stratified prevalence of current smoking, alcohol drinking, physical inactivity, and obesity in both men and women. Among these parameters, men exhibited a notably higher prevalence of smoking and alcohol drinking across all age categories, while women displayed a greater prevalence of physical inactivity and obesity than men. An observable decline in the prevalence of current smoking was evident with advancing age in men, extending across all four regions, except for the age groups of 40 years in Aktobe and 45 years in Atyrau, with similar exceptions identified for alcohol drinking among men aged 40–55, except in the Atyrau region among 45-year-old men. Conversely, a discernible trend indicated an elevated prevalence of physical inactivity among all participants as age increased. Additionally, the prevalence of obesity demonstrated an upward trajectory with age, particularly among urban men aged 45–55 years.

### 3.3 Gender-related prevalence of NCDs in West Kazakhstan

**Figure 2:**
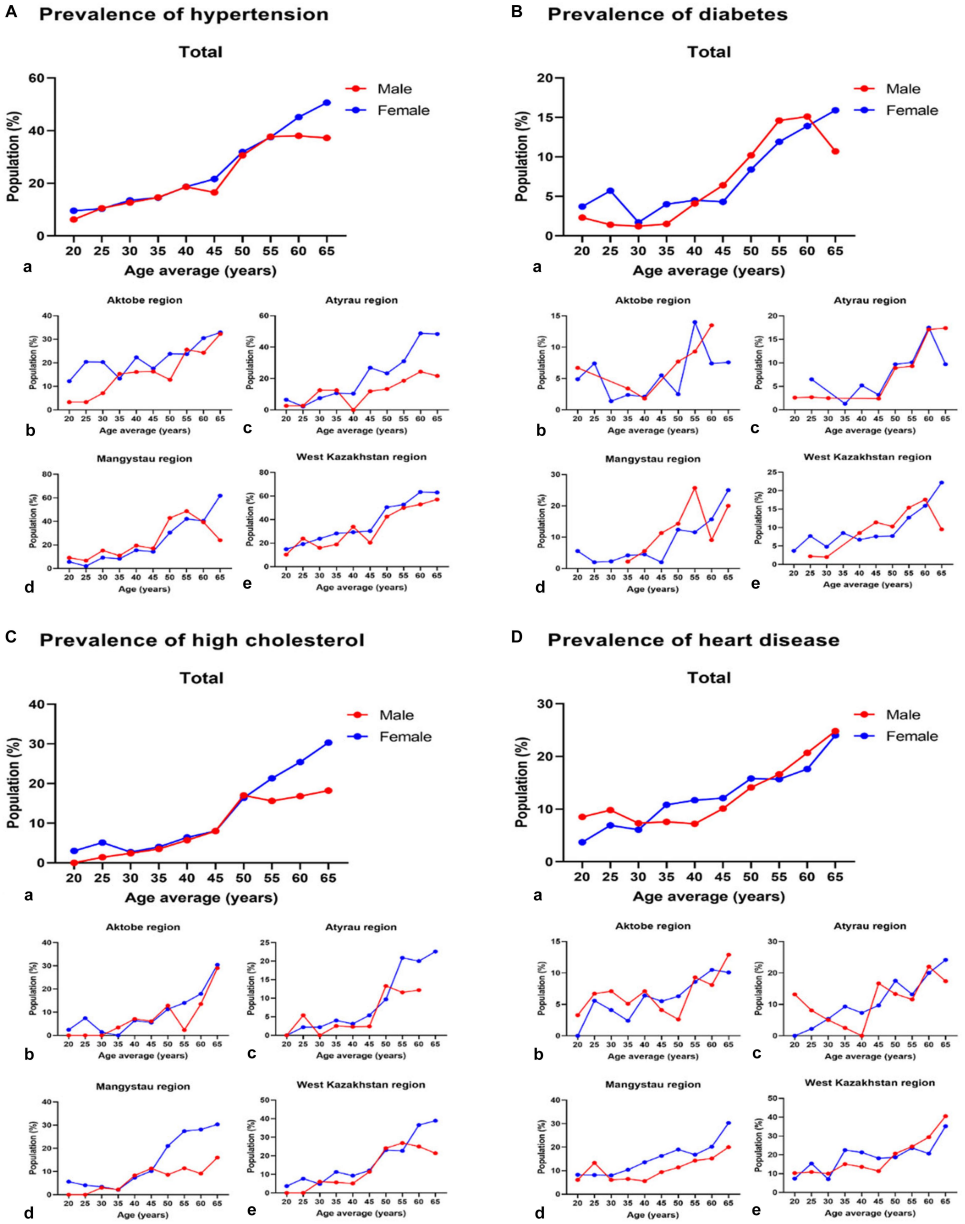
Prevalence of hypertension, diabetes, high cholesterol, and heart disease among men and women in the regions of West Kazakhstan. a–e Graphs with small letters labelling show total and four regions of west Kazakhstan.

Figure 2 depicts the age-specific prevalence of hypertension and diabetes in both men and women across four regions in West Kazakhstan. The data reveal a considerably elevated prevalence of hypertension, diabetes, high cholesterol, and heart disease in both men and women across all age groups, with the only exception being diabetic women in the Aktobe and Atyrau regions within the age range of 60–69 years. Additionally, a higher prevalence of high cholesterol was observed in men, and a greater prevalence of heart disease was identified in women.

### 3.4 Regional prevalences of smoking, alcohol drinking, physical inactivity, and obesity in West Kazakhstan

The regions of West Kazakhstan and Mangistau displayed the highest prevalence of smoking, at 21.0% (95%CI: 8.0–34.0%) and 17.8% (95%CI: 5.8–29.7%), respectively. Notably, the three regions with the highest prevalence of alcohol drinking were the West Kazakhstan region (64.0, 95%CI: 41.4–86.6%), the Mangystau region (52.9, 95%CI: 32.4–73.4%), and the Aktobe region (43.3, 95%CI: 24.8–61.9%). Furthermore, the Aktobe and Mangistau regions were distinguished by having the highest prevalence of physical inactivity, with rates of 89.7% (95%CI: 63.0–116.3%) and 88.9% (95%CI: 62.4–115.5%), respectively. The West Kazakhstan region, particularly in the northern part, exhibited a relatively high prevalence of obesity at 24.7% (95%CI: 10.6–38.7%). Regarding the prevalence of hypertension, diabetes, high cholesterol, and heart disease, the West Kazakhstan region recorded the highest rates than the other regions, with respective percentages of 39.0% (95%CI: 21.4–56.6%), 9.7% (95%CI: 0.9–18.5%), 17.3% (95%CI: 5.5–29.0%), and 20.0% (95%CI: 7.4–32.6%). Additional detailed information is shown in Supplementary Table S1.

**Figure 3:**
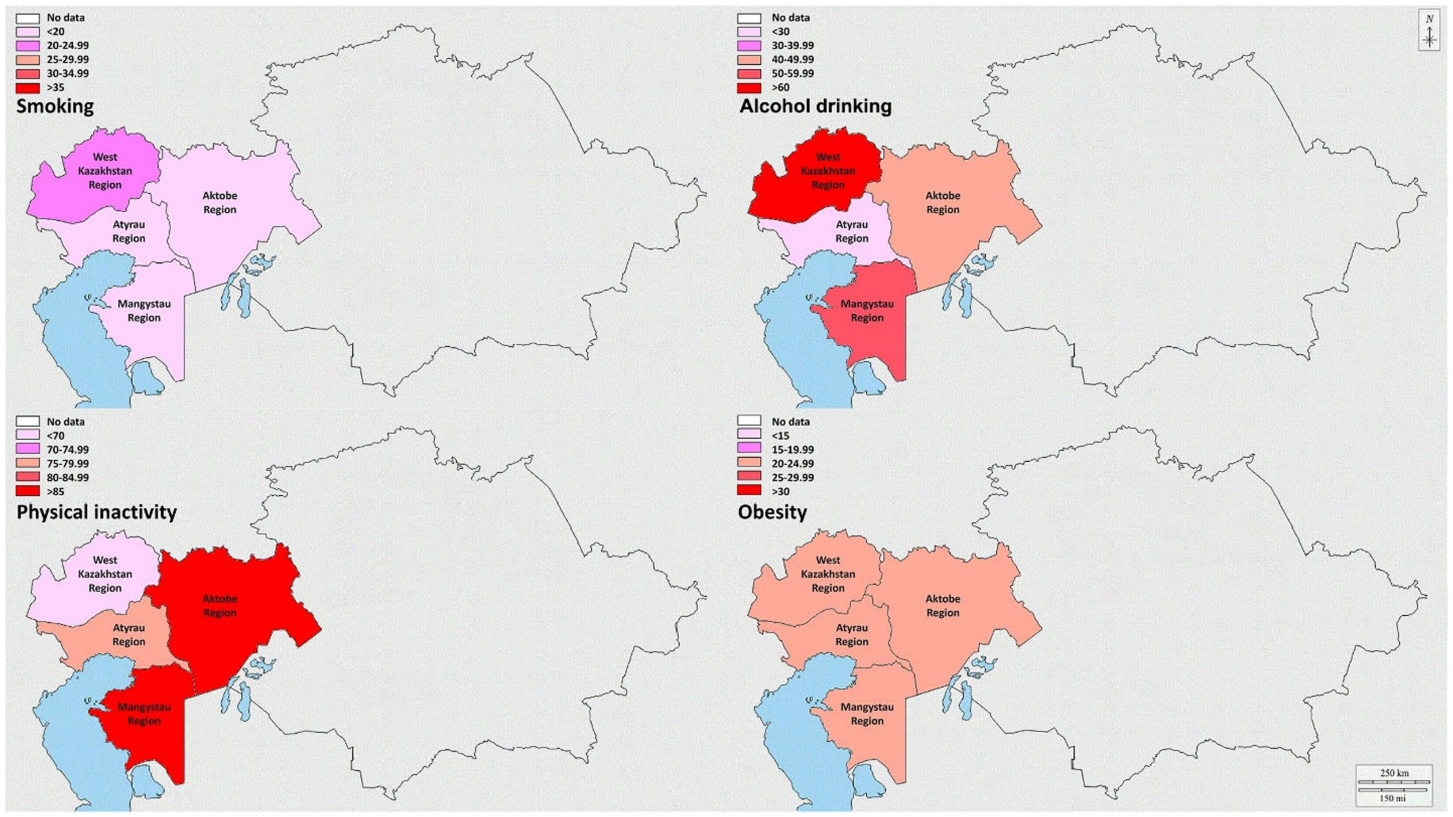
Prevalence of smoking, alcohol drinking, physical inactivity, and obesity in the regions of West Kazakhstan.

Figure 3 depicts the prevalence of behavioral risk factors in a particular region. Furthermore, Supplementary materials present an illustration of the prevalence of these behavioral risk factors for both genders across the four regions in West Kazakhstan (Supplementary Figure S1), respectively.

### 3.5 Regional prevalences of NCDs in West Kazakhstan

**Figure 4:**
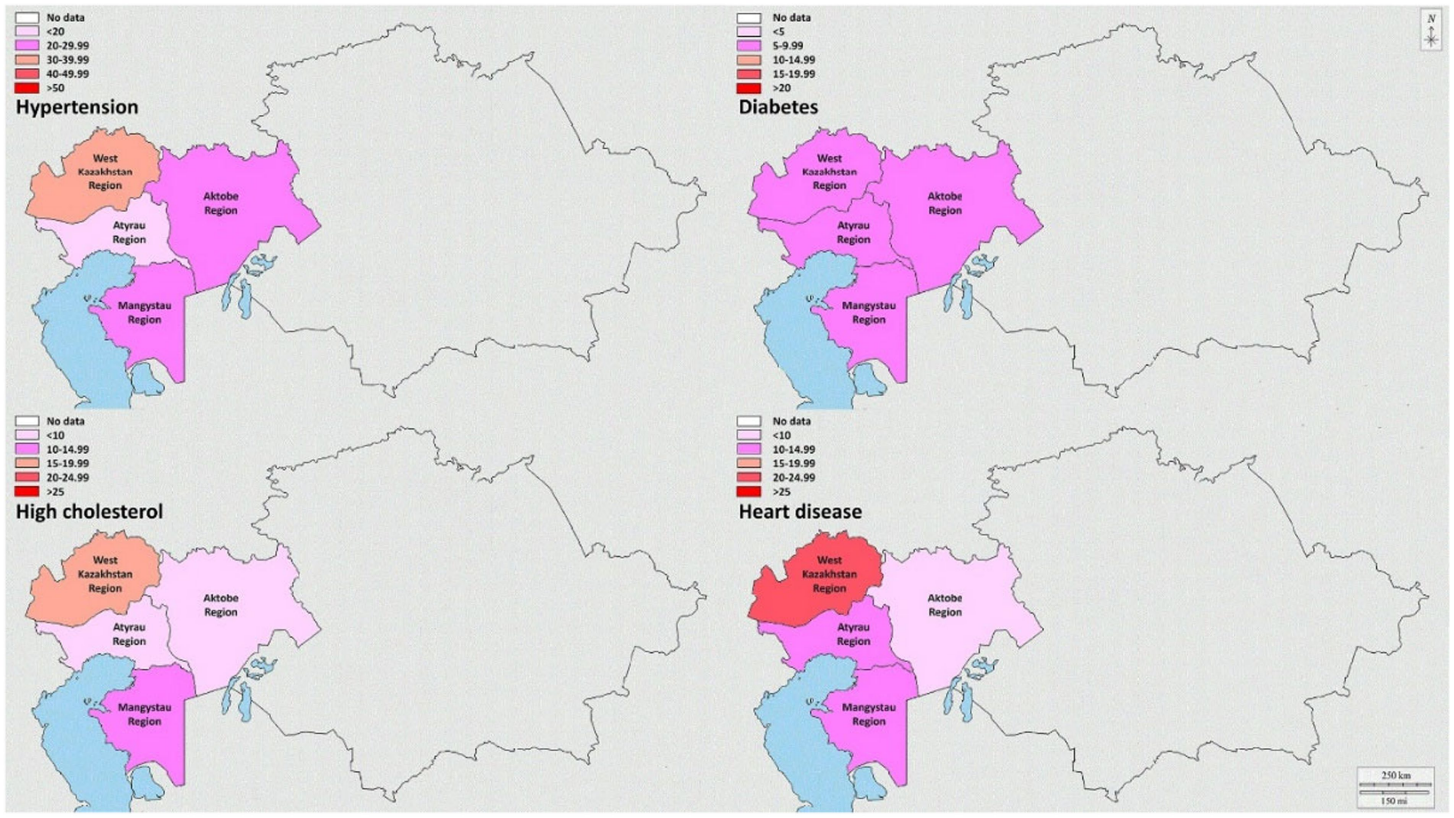
Prevalence of hypertension, diabetes, high cholesterol, and heart disease in the regions of West Kazakhstan.

Figure 4 shows the comprehension of NCD prevalence within the four regions of West Kazakhstan. Additionally, the Supplementary materials offer a visual representation of the prevalence of NCDs in both men and women across the four regions in West Kazakhstan (Supplementary Figure S2), separately.

### 3.6 Association of prevalences of NCDs and smoking, alcohol drinking, physical activity, and obesity in West Kazakhstan

The results from the multinomial logistic regression analysis indicated a significant association between increased physical activity and a reduced risk of hypertension (odds ratio [OR]: 0.704; 95% confidence interval [CI]: 0.593–0.837), diabetes (OR: 0.647; 95%CI: 0.494–0.846), and heart disease (OR, 0.514; 95%CI: 0.419–0.630). In contrast, obesity exhibited a significant association with a heightened risk of hypertension (OR: 1.574; 95%CI: 1.362–1.819), high cholesterol (OR: 1.705; 95%CI: 1.403–2.072), and heart disease (OR: 1.414; 95%CI: 1.178–1.697). However, neither alcohol drinking nor smoking displayed significant associations with any of the examined metabolic conditions (Table 4).

**Table 4:** Association between behavioral risk factors and metabolic conditions.

Index	Hypertension	Diabetes	High cholesterol	Heart disease
Smoking	1.264 (1.021–1.566) *	1.059 (0.751–1.494)	0.973 (0.718–1.318)	1.327 (1.027–1.714) *
Alcohol drinking	0.889 (0.756–1.045)	0.961 (0.741–1.246)	0.907 (0.731–1.125)	1.010 (0.821–1.241)
Physical inactivity	0.704 (0.593–0.837) ***	0.647 (0.494–0.846) ***	0.711 (0.594–0.896) **	0.514 (0.419–0.630) ***
Obesity	1.574 (1.362–1.819) ***	1.235 (0.980–1.557)	1.705 (1.403–2.072) ***	1.414 (1.178–1.697) ***

Multinomial logistic regression was used to estimate the odds ratio (95% confidence interval) of metabolic conditions in relation to smoking, alcohol drinking, physical inactivity, and obesity. Models were adjusted for age, gender, education and living region, ethnicity, marital status, labor force status, and fruit and vegetable consumption. *p < 0.05; **p < 0.01; ***p < 0.001.

The inclusion of Table 4 serves to explore the nuanced relationship between behavioral risk factors and metabolic risk factors, taking into account the potential moderating effect of gender. While it is acknowledged that the association between behavioral and metabolic risk factors has been observed across various cultural settings, our study aims to specifically investigate whether sex differences modulate this relationship.

The decision to adjust for gender in the final analysis is rooted in the objective of understanding how gender may influence the associations between behavioral and metabolic risk factors. By treating gender as a potential moderator, we aim to discern whether the impact of behavioral risk factors on metabolic outcomes differs between men and women. This approach allows for a more granular exploration of sex differentials in the interplay between lifestyle choices and metabolic health.

The adjustment for gender in Table 4 enables us to delineate whether the observed variations in behavioral risk factors translate into similar or distinct metabolic risk factor prevalence among men and women, providing a valuable perspective on whether sex-specific patterns exist in the relationship between behavior and metabolic health outcomes. This adjustment enhances the depth of our analysis, contributing to a more comprehensive understanding of gender-related disparities in the studied population. The choice to present gender-adjusted results aligns with the study’s focus on sex differentials and allows for the identification of potential variations in the associations that may be obscured in unadjusted analyses.

## 4 Discussion

Our analysis of West Kazakhstan regions unveiled significant findings, including the overall prevalence rates of behavioral risk factors and metabolic conditions: smoking (13.6%), alcohol drinking (47.0%), current obesity (22.3%), and physical inactivity (80.7%). Furthermore, NCDs showed an overall prevalence rate, including hypertension (25.6%), diabetes (7.5%), high cholesterol (11.8%), and heart diseases (13.0%). Key insights are as follows: (1) This study exposed varying prevalence rates of essential behavioral risk factors and NCDs among West Kazakhstan’s population. Notably, high levels of physical inactivity and obesity were identified, affecting a substantial portion of the populace. (2) Gender disparities manifested in smoking, alcohol drinking, physical inactivity, and obesity. Men displayed higher smoking and alcohol drinking rates, while women exhibited a greater prevalence of physical inactivity and obesity. (3) Region-specific analysis unveiled distinct patterns of risk factors and NCDs, with the West Kazakhstan region reporting the highest prevalence of smoking, alcohol drinking, and specific metabolic conditions. These findings underscore the significance of tailored public health interventions at the regional level.

The findings of this study shed light on the prevalent behavioral risk factors and metabolic conditions among the population of West Kazakhstan, offering insights into the region’s public health landscape. These results prompt discussions on multiple levels, including the implications for public health interventions, the effect of gender disparities, and the importance of region-specific approaches (13, 14).

The study’s primary outcome, the prevalence of behavioral risk factors and metabolic conditions, reveals several notable trends. Smoking, alcohol drinking, obesity, and physical inactivity were identified as common concerns in West Kazakhstan. These factors pose significant challenges to the population’s health and well-being, necessitating comprehensive public health strategies to address them effectively (3, 15–17).

The study also illuminates distinct gender disparities in the prevalence of risk factors and conditions. Men exhibited higher rates of smoking and alcohol drinking, underscoring the need for targeted interventions to reduce these behaviors among men. On the other hand, women displayed a greater prevalence of physical inactivity and obesity. Understanding these gender-specific differences is crucial for developing interventions tailored to the specific needs of both men and women (18–20).

The region-specific analysis revealed variations in the patterns of risk factors and NCDs across West Kazakhstan. The West Kazakhstan region, notably the northern part, displayed higher rates of smoking, alcohol drinking, and specific metabolic conditions. This finding emphasizes the importance of tailoring public health interventions to address the unique challenges faced by each region. Localized efforts may include region-specific health campaigns, community engagement, and targeted healthcare services (21, 22).

The results of the multinomial logistic regression analysis provide valuable insights into the relationship between risk factors and metabolic conditions. Notably, physical inactivity exhibited a significant association with an increased risk of hypertension, diabetes, and heart disease. Conversely, obesity was significantly associated with a heightened risk of hypertension, high cholesterol, and heart disease. While the absence of significant associations between smoking and alcohol drinking with these conditions may appear counterintuitive, further research is required to understand the intricate relationships between these factors and metabolic health fully (23–25).

This study offers a comprehensive view of the behavioral risk factors and metabolic conditions prevalent in West Kazakhstan. Understanding these patterns, gender disparities, and regional variations is pivotal for the development of targeted public health interventions aimed at improving the health and well-being of the region’s population. Further research is needed to explore the complex interactions between these risk factors and conditions, enabling the design of more effective and tailored interventions.

## 5 Limitations

While our study contributes valuable insights into the prevalence of behavioral risk factors and NCDs in West Kazakhstan, it is essential to acknowledge several limitations that may impact the interpretation of our findings.

Cross-Sectional Design: The cross-sectional nature of our study design limits our ability to establish causal relationships between behavioral risk factors, metabolic conditions, and demographic variables. Longitudinal studies would be more suitable for exploring temporal trends and causal associations over time.

Self-Reported Data: The reliance on self-reported data, particularly for lifestyle behaviors such as smoking, alcohol drinking, and physical activity, introduces the potential for recall bias. Participants may underreport or overreport certain behaviors due to social desirability or memory lapses.

Generalization to Other Regions: Our study focuses on the West Kazakhstan regions, and caution should be exercised when generalizing the findings to other geographical areas. Variations in cultural, socioeconomic, and healthcare factors may influence the prevalence rates of behavioral risk factors and NCDs in different regions.

Exclusion of Specific Age Groups: While the chosen age range (18–69 years) provides a comprehensive view of the adult population, the exclusion of individuals outside this range limits our understanding of the prevalence of behavioral risk factors and NCDs in younger and older age groups.

Limited Exploration of Additional Risk Factors: Our study primarily focuses on key behavioral risk factors and metabolic conditions. While these factors are crucial, the exclusion of other potential risk factors, such as genetic predispositions and environmental exposures, limits the comprehensive assessment of determinants influencing health outcomes.

BMI Calculation Methodology: Although efforts have been made to enhance the clarity of our BMI calculation methodology, it is important to note that the accuracy of BMI values relies on precise weight and height measurements. Variations in measurement techniques or instruments may introduce minor inaccuracies.

Limited Socioeconomic and Cultural Context: The study lacks an in-depth exploration of the socioeconomic and cultural factors that may influence behavioral risk factors and NCDs. A more comprehensive understanding of the broader context would provide additional insights into the observed patterns.

Sampling Methodology: While our sampling strategy aimed at achieving a representative sample, the use of cluster sampling introduces the potential for sampling bias. Variability within selected clusters may impact the generalizability of our findings to the entire West Kazakhstan population.

In light of these limitations, readers are encouraged to interpret our findings with caution, recognizing the inherent constraints of our study design and methodology. Addressing these limitations in future research endeavors will contribute to a more nuanced understanding of the complex interplay between behavioral risk factors, NCDs, and the unique contextual factors influencing health outcomes in the West Kazakhstan region.

## 6 Conclusion

In conclusion, this study conducted in West Kazakhstan revealed significant variations in the prevalence of behavioral risk factors and NCDs across different regions, genders, and age groups. Notable findings include higher rates of smoking and alcohol drinking in men, while women exhibited a greater prevalence of physical inactivity and obesity. The prevalence of these risk factors and metabolic conditions varied by region, with the West Kazakhstan region demonstrating distinct patterns, including higher rates of smoking, alcohol drinking, physical inactivity, obesity, and metabolic conditions. Moreover, the study highlighted the importance of addressing these risk factors and tailoring interventions to address specific demographic and regional characteristics to improve public health in West Kazakhstan, considering variability in genders.

## Data availability statement

The raw data supporting the conclusions of this article will be made available by the authors, without undue reservation.

## Ethics statement

The studies involving humans were approved by Local Ethics Committee of S.D. Asfendiyarov Kazakh National Medical University, Almaty, Republic of Kazakhstan. The studies were conducted in accordance with the local legislation and institutional requirements. The participants provided their written informed consent to participate in this study.

## Author contributions

AB: Conceptualization, Data curation, Formal analysis, Funding acquisition, Investigation, Methodology, Project administration, Software, Supervision, Writing – original draft. AA: Data curation, Investigation, Writing – review & editing. NM: Methodology, Resources, Validation, Writing – review & editing. AK: Methodology, Resources, Validation, Visualization, Writing – review & editing. GY: Methodology, Software, Writing – review & editing. SZ: Investigation, Methodology, Writing – review & editing. ZA: Investigation, Methodology, Writing – review & editing. KZ: Investigation, Methodology, Validation, Writing – review & editing. AU: Formal analysis, Methodology, Validation, Writing – review & editing. AN: Investigation, Methodology, Validation, Writing – original draft, Writing – review & editing. AT: Conceptualization, Data curation, Formal analysis, Investigation, Methodology, Project administration, Software, Supervision, Writing – original draft.
