# Supplementary material for: Sex differentials in the prevalence of behavioral risk factors and non-communicable diseases in adult populations of West Kazakhstan
Source: Front Public Health. 2024 Feb 14;12:1333887. doi: 10.3389/fpubh.2024.1333887 (PMC10899439; doi:10.3389/fpubh.2024.1333887)
Supplement: Supplementary file 1 [file Table_1.DOCX]

Supplementary Material

# Supplementary Figures and Tables

## Supplementary Tables

**Table S1.** Geographic prevalence (95% confidence interval) of smoking, drinking, physical inactivity and obesity

| Variables | Aktobe region | Atyrau region | West Kazakhstan region | Mangystau region |
| --- | --- | --- | --- | --- |
| Current smoking | 8,5 (0.3-16.7) | 7.3 (-0.3-15.0) | 21.0 (8.0-34.0) | 17.8 (5.8-29.7) |
| Alcohol drinking | 43.3 (24.8-61.9) | 27.6 (12.7-42.4) | 64.0 (41.4-86.6) | 52.9 (32.4-73.4) |
| Physical inactivity | 89.7 (63.0-116.3) | 64.9 (42.2-87.6) | 79.4 (54.3-104.5) | 88.9 (62.4-115.5) |
| Obesity | 20.1 (7.4-32.7) | 22.2 (8.9-35.5) | 24.7 (10.6-38.7) | 22.1 (8.8-35.4) |
| Hypertension  Diabetes  High cholesterol  Heart disease | 20.1 (7.4-32.7)  5.1 (-1.3-11.5)  9.1 (0.6-17.6)  6.4 (-0.7-13.6) | 19.3 (6.9-31.8)  6.5 (-0.7-13.7)  8.5 (0.3-16.7)  11.4 (1.9-21.0) | 39.0 (21.4-56.6)  9.7 (0.9-18.5)  17.3 (5.5-29.0)  20.0 (7.4-32.6) | 23.9 (10.1-37.7)  8.7 (0.3-17.0)  12.3 (2.4-22.3)  14.1 (3.5-24.7) |

## Supplementary Figure


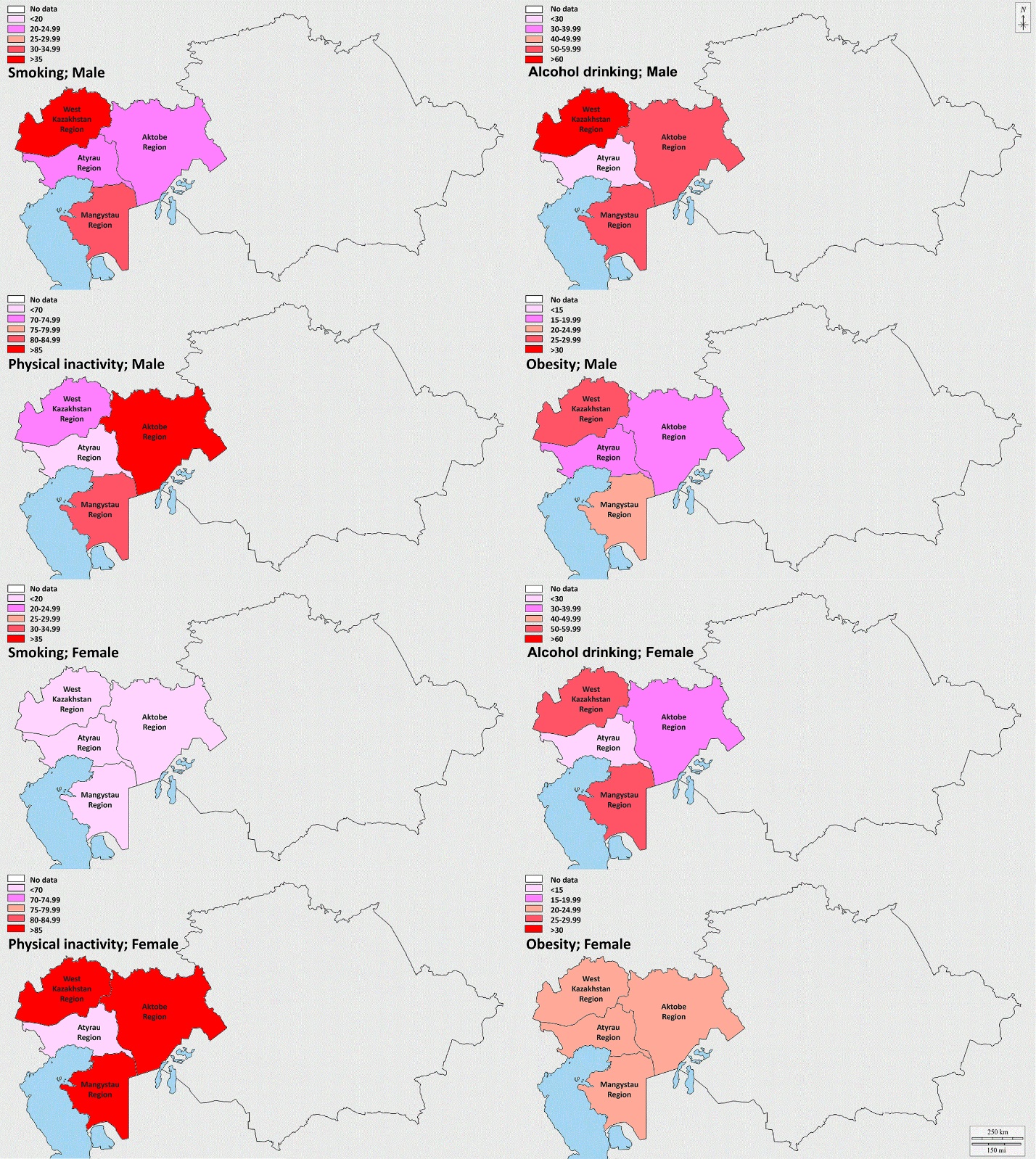


**Figure S1.** Prevalence of smoking, alcohol drinking, physical inactivity and obesity by male and female in the regions of Western Kazakhstan.

**
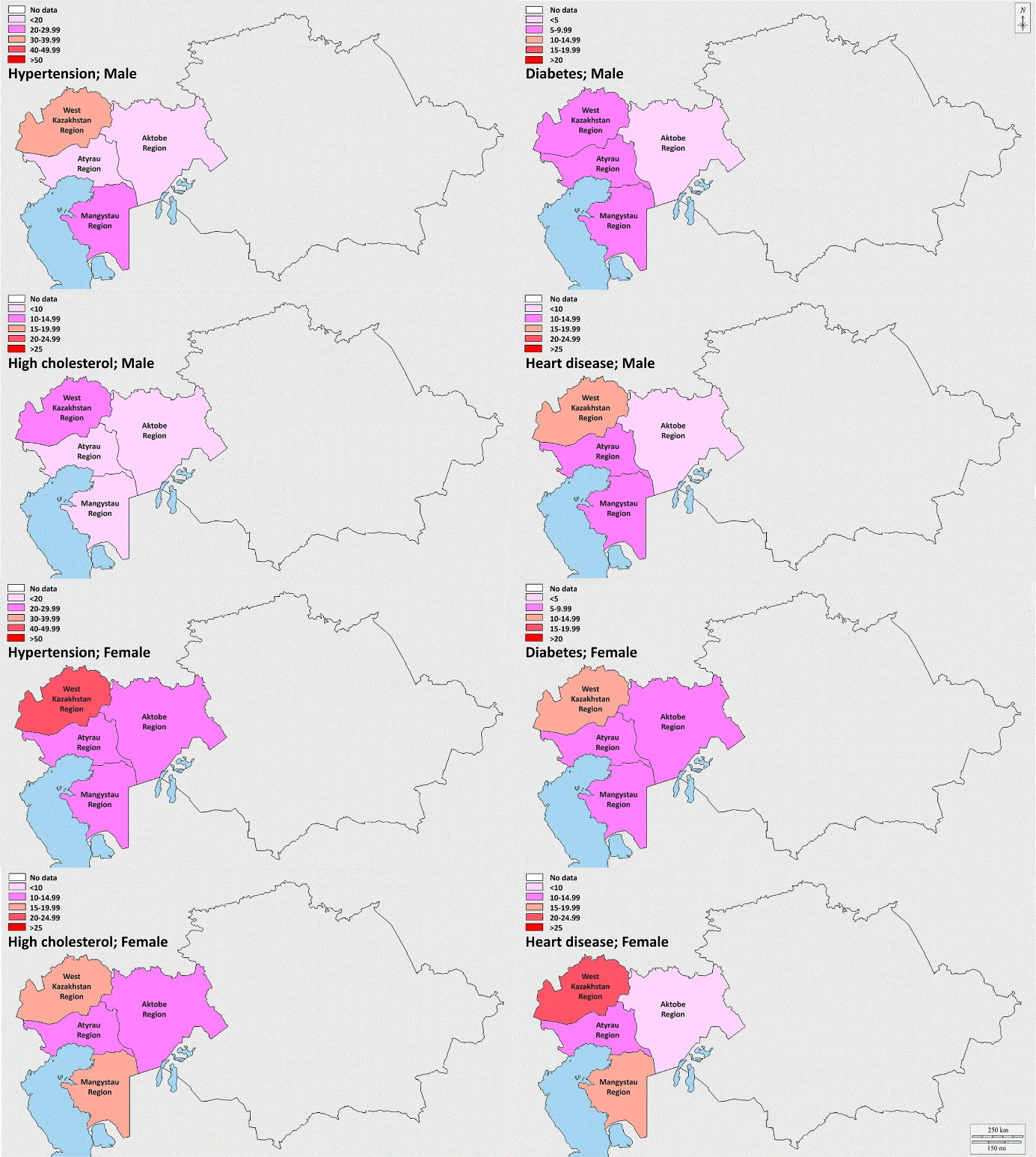
**

**Figure S2.** Province-specific prevalence of hypertension, diabetes, high cholesterol and heart disease by male and female in the regions of Western Kazakhstan
